# Structure of the Capsid Size-Determining Scaffold of “Satellite” Bacteriophage P4

**DOI:** 10.3390/v12090953

**Published:** 2020-08-27

**Authors:** James L. Kizziah, Cynthia M. Rodenburg, Terje Dokland

**Affiliations:** Department of Microbiology, University of Alabama at Birmingham, Birmingham, AL 35294, USA; kizziah4@uab.edu (J.L.K.); crodenburg@fa.ua.edu (C.M.R.)

**Keywords:** *Caudovirales*, mobile genetic elements, molecular piracy, bacteriophage P2, capsid assembly, size determination, Psu, Sid, *sir* mutants

## Abstract

P4 is a mobile genetic element (MGE) that can exist as a plasmid or integrated into its *Escherichia coli* host genome, but becomes packaged into phage particles by a helper bacteriophage, such as P2. P4 is the original example of what we have termed “molecular piracy”, the process by which one MGE usurps the life cycle of another for its own propagation. The P2 helper provides most of the structural gene products for assembly of the P4 virion. However, when P4 is mobilized by P2, the resulting capsids are smaller than those normally formed by P2 alone. The P4-encoded protein responsible for this size change is called Sid, which forms an external scaffolding cage around the P4 procapsids. We have determined the high-resolution structure of P4 procapsids, allowing us to build an atomic model for Sid as well as the gpN capsid protein. Sixty copies of Sid form an intertwined dodecahedral cage around the *T* = 4 procapsid, making contact with only one out of the four symmetrically non-equivalent copies of gpN. Our structure provides a basis for understanding the *sir* mutants in gpN that prevent small capsid formation, as well as the *nms* “super-sid” mutations that counteract the effect of the *sir* mutations, and suggests a model for capsid size redirection by Sid.

## 1. Introduction

True to his principle of studying what is interesting rather than what is fashionable, Michael Rossmann initiated his study of bacteriophage structure in the early 1990s, at a time when phage research had long been in decline. His pioneering research on two of the most classic systems in phage research—ϕX174 and T4—was a major contributor to the subsequent revival of the field. The single-stranded DNA virus ϕX174—the type member of the *Microviridae* family of phages—is an unusual phage in that it assembles its procapsid using an external scaffold [1,2], rather than the internal scaffolding proteins used by the more well-known double-stranded (ds) DNA phages of the *Caudovirales*, such as λ, T4 and P2 [3]. As it turns out, the *Microviridae* are not completely unique in this respect: Around the same time that Michael initiated his ϕX174 project, “satellite” phage P4 was found to use an external scaffold to re-direct the assembly pathway of its “helper” phage P2 [4].

Bacteriophage P2, a tailed phage of the family *Myoviridae* in order *Caudovirales* is a temperate bacteriophage with a 33.6 kb dsDNA genome [5]. Its virion consists of a ≈60 nm diameter icosahedral capsid and a 135 nm long contractile tail, tipped with a baseplate and six tail fibers [6,7]. P2 was originally isolated from *Escherichia coli* and is the prototypical member of the P2-like prophages, which are abundant among the Gammaproteobacteria [5,8]. P2 procapsids are assembled from pentamers and hexamers of the major capsid protein, gpN on a *T* = 7 *dextro* icosahedral lattice [9], together with the internal scaffolding protein, gpO [7], and a dodecamer of the portal protein (traditionally called “connector”), gpQ, at a single, unique capsid vertex [10,11]. During maturation, gpN, gpO, and gpQ are proteolytically cleaved, presumably by gpO, which includes an N-terminal protease domain [7,12,13]. Packaging of the dsDNA genome requires the terminase proteins, gpM and gpP [14]. A head completion protein, gpL, is added [7], and the tail is attached to the capsid to complete the P2 virion.

“Satellite” phage P4 is not a true phage, but rather a “pirate” mobile genetic element (MGE) [15,16]—an integrative plasmid that has acquired the ability to utilize a “helper” phage, such as P2, for its own propagation [17]. When P2 infects a cell harboring a P4 element, *trans*-activation of P4 by the P2 transcriptional activator Ogr leads to P4 excision (if integrated), replication, and expression of genes involved in *trans*-regulation of P2, including δ, *psu* and *sid* [17]. Delta is a transcriptional activator and a homolog of Ogr [18], while Psu is a suppressor of polar amber mutants in P2 [19], and also acts as a stabilizing “decoration” protein that binds to the outside of P4 capsids [20,21,22,23]. Ultimately, P4 genomes become packaged into phage particles made primarily from P2-encoded structural proteins. However, the Sid (“Size determination”) protein encoded by P4 changes the P2 capsid from its normal 60 nm diameter, *T* = 7 organization to a 45 nm capsid with *T* = 4 architecture [4,6,9,24]. The smaller capsid fits the 11.6 kb P4 genome, but is too small to package the 33.6 kb P2 genome, thereby leading to strong suppression of P2 burst size. The same process occurs if the P4 particles infect a cell harboring a P2 prophage, in which case the P4-encoded Epsilon protein leads to derepression of the prophage, followed by the same process described above [25]. Mutants in the gpN capsid protein (called *sir* for “size responsiveness”) were previously found that blocked the ability of Sid to form small capsids [26]. Similarly, mutations in Sid (*nms* or “super-sid” mutants) were identified that enabled P4 to form small capsids even in a *sir* mutant [27].

We previously solved the structures of the P2 and P4 procapsids to 8–9 Å resolution using cryo-electron microscopy (cryo-EM) and icosahedral reconstruction [9]. These structures showed the expected HK97-like fold of the gpN capsid protein and revealed that the *sir* mutations were clustered at the apex of the A-domain, where the gpN–Sid interaction occurred. However, the resolution was too low to fully model gpN. The Sid scaffold appeared to have a mostly α-helical organization, but due to the low resolution and the lack of an existing model for Sid, the protein could not be modeled into the density. These reconstructions used relatively small datasets (4000–8000 particles) made from images collected on photographic film, before the advent of direct electron detectors and the availability of more stable, automated microscopes. We have now collected a much larger dataset of >40,000 particles using a Titan Krios microscope equipped with a Gatan K3 direct electron detector, allowing an asymmetric reconstruction of the P4 procapsid to be calculated to near-atomic resolution. This structure shows unambiguously the fold of the Sid protein and its interaction with gpN, suggests a model for Sid action, and explains the role of *sir* and *nms* mutations in blocking and restoring P2 capsid size redirection by P4.

## 2. Materials and Methods

### 2.1. Production of P4 Procapsids

**Figure 1:**
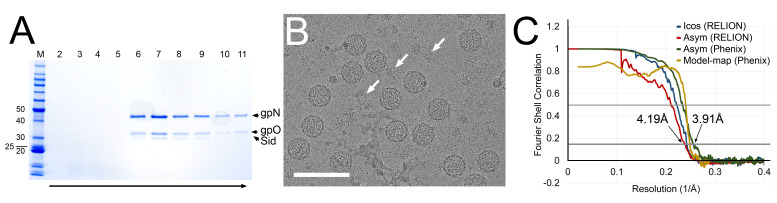
(**A**) SDS-PAGE from sucrose gradient purification of P4 procapsids. The lanes are labeled by fraction number, and the arrow shows the direction of sedimentation. Positions of bands corresponding to gpN, gpO and Sid are indicated. M = marker, with pertinent molecular weights (kDa) listed. (**B**) Cryo-electron micrograph of P4 procapsids. The arrows point to additional proteinaceous material assumed to be Sid. Scale bar, 100 nm. (**C**) Fourier Shell Correlation (FSC) plots for the icosahedral (blue) and asymmetric (red) reconstructions calculated in RELION, for the asymmetric map calculated in Phenix (green) and the correspondence between the atomic model and the reconstruction (yellow), calculated in Phenix. The gray lines show the 0.143 and 0.5 cutoff levels.

The protease-deficient mutant gpO protein, O(S107A) was previously described [12], as was the pET16-based plasmid pJRC49 for co-expression of O(S107A) and gpN, yielding P2 procapsids [12]. Here, the P4 *sid* gene expressing full-length Sid protein was added, including its own ribosome binding site, after the *N* gene in pJRC49, yielding plasmid pCMR22. The plasmid was introduced into *E. coli* BL21(DE3), and the cells were grown in LB + ampicillin and induced with 1 mM IPTG at A_600_ = 0.9 OD. Cells were grown for 2 h, harvested by centrifugation at 6,000× *g* for 10 min, resuspended in lysis buffer (100 mM Tris-HCl pH 7.4, 200 mM NaCl, 10 mM MgCl_2_, 1 mM PMSF, 1% Triton X-100 and 0.5% sodium deoxycholate) and lysed by two cycles of freezing/thawing, followed by addition of 1 µL (≈250 units) Benzonase^®^ nuclease (Sigma-Aldrich, St. Louis, MO, USA) and three passes through an Avestin Emulsiflex B15 high-pressure cell disruptor (Avestin, Ottawa, ON, Canada). After clarification of the lysate at 12,000× *g* for 30 min, the supernatant was pelleted at 40,000× *g* for 1 h and resuspended in 5 mL procapsid buffer (100 mM Tris pH 7.4, 200 mM NaCl, 10 mM MgCl_2_). The suspension was loaded on a 5–30% sucrose gradient in procapsid buffer, centrifuged at 30,000 RPM in a Beckman SW41 rotor for 2 h, and fractionated into 13 1-mL fractions. Fractions 7–11, containing gpN, gpO and Sid (Figure 1A), were pooled, pelleted, and resuspended in 20 mM Tris, 50 mM NaCl, 2 mM MgCl_2_.

### 2.2. Cryo-Electron Microscopy

Cryo-EM samples were made as previously described, using a Vitrobot Mark IV and glow-discharged nickel Quantifoil R2/2 grids [9]. Cryo-EM data were collected at the Southeastern Consortium for Microscopy of Macromolecular Machines (SECM4) at Florida State University, using a Titan Krios microscope (Thermo Fisher Scientific, Waltham, MA, USA) operating at 300 kV, and a Gatan K3 detector (Gatan Inc., Pleasanton, CA, USA) mounted post-Gatan imaging filter. Images were collected without energy filtering at a magnification of 45,000× (1.11 Å per pixel), 0.5 to 2.5 µm defocus, and a total dose of 30 e^−^/Å^2^ (Figure 1B; Table S1).

### 2.3. Three-Dimensional Reconstruction and Model Building

RELION-3.0.7 [28] was used throughout the data processing and reconstruction process (Figure S1). After motion correction with dose weighting in MotionCor2 [29] and CTF determination using Gctf v1.06 with equi-phase averaging [30], both within RELION-3, a total of 6989 micrographs were used for particle picking, after removing micrographs with defocus greater than 4 µm, estimated maximum resolution worse than 5 Å, or otherwise aberrant CTF estimates. Two 2D class averages generated from 406 manually picked P4 procapsid particles were used as references for autopicking 241,695 particles. After particle extraction with six-fold binning (6.66 Å/pixel), the data set was reduced to 156,395 particles through iterative 2D classification with a 550 Å diameter mask. A 3D model was generated de novo via stochastic gradient descent assuming icosahedral symmetry and used to initiate iterative 3D classification without application of symmetry (Figure S1). The data set was reduced to 44,508 particles belonging to classes with the best Sid density. After switching to the unbinned (1.11 Å/pixel) data, auto-refinement assuming icosahedral symmetry using a 740 Å mask resulted in a map at 4.13 Å resolution by the 0.143 FSC criterion (Figure 1C; Table S1). However, the density for the Sid protein scaffold was poor, precluding modeling of Sid.

The refined particles were re-extracted with three-fold binning (3.33 Å/pixel) and symmetry-expanded using *relion_particle_symmetry_expand* according to icosahedral symmetry [31]. The icosahedral reconstruction was segmented using Segger in UCSF Chimera [32]. External scaffold density stretching from one three-fold symmetry axis to another via the two-fold axis was used to create a soft mask. Two rounds of asymmetric, masked 3D classification with a 550 Å mask removed any symmetry-expanded particles without Sid density in the masked area, leaving 438,018 particles. These particles were re-extracted without binning (1.11 Å/pixel) and auto-refined without imposing symmetry with a larger mask incorporating the 3D-classified Sid density and the surrounding capsomers of gpN to a resolution of 3.91 Å (Figure 1C; Table S1). In this map, individual copies of Sid and gpN could be clearly distinguished and traced using bulky amino side chains to guide the atomic model building. Global FSCs were calculated in RELION-3 postprocessing and phenix.validation_cryoEM, and local FSCs were calculated in ResMap v1.1.4 [33] (Figure S2). Maps were rendered in Chimera at 5σ above the mean, unless noted.

### 2.4. Model Building and Refinement

The full-length amino acid sequences for gpN (NCBI Accession number: NP_046760) and Sid (NP_042042) were input to I-TASSER [34] to generate initial atomic models. After fitting of the highest scoring I-TASSER model of gpN into the reconstruction in UCSF Chimera, the model was manually modified by adjusting the relative positions of secondary structure elements, repositioning and retracing random coils, and removing any residues not well-defined in the density using *Coot* v0.8.9.2 [35]. The manually corrected model was then copied to the remaining unique gpN positions and adjusted further as needed. The I-TASSER models for Sid could not be fitted directly into the reconstruction. Instead, several helical segments of the highest scoring model were placed based on secondary structure transitions and bulky amino acid side chain density then extended through the remainder of the Sid density.

The atomic models were refined through iterative cycles of global real-space refinement with phenix.real_space_refine and local refinement and adjustment in *Coot* [33,35]. Surrounding icosahedral symmetry-related copies of Sid and gpN were generated in UCSF Chimera and included for intermolecular context during real-space refinement. To reduce computation hours, the P4 procapsid reconstruction was zoned to within 40 pixels of the complete model (including context), cropped to 320^3^ pixels, and re-centered. Automated refinement weight estimation was included in early cycles in phenix.real_space_refine, while a weight of 3 was used for later runs. Secondary structure restraints generated from the input models, rotamer restraints, and Ramachandran restraints were included throughout. Local refinement included automatically estimated weight matrices and Ramachandran, torsion, and peptide bond restraints. The model-to-map FSC and cross-correlations were calculated in phenix.validation_cryoem [33]. The final model was validated using MolProbity and EMRinger and by comparing model-to-half map FSCs calculated in Mtriage [33,36,37] (Table S2). The final model without context was translated back into position in the unaltered P4 procapsid reconstruction prior to deposition (EMDB ID: EMD-22513 and PDB ID: 7JW1).

## 3. Results

### 3.1. Structure Determination

P4 procapsids were generated by *E. coli* co-expression of gpN, Sid and a protease-deficient form of gpO [O(S107A)], purified on sucrose gradients (Figure 1A), and prepared for cryo-EM by standard methods [12]. A total of 156,395 particles with the typical thick-shelled morphology of procapsids were picked from 6989 images (Figure 1B), and processed using RELION-3. Capsids that were obviously large or misshapen were excluded from the analysis. Subsequent 3D classification revealed that ≈29% (44,508 particles) of the particles consisted of procapsids with external scaffolding, while ≈71% of the particles lacked the external scaffold (Figure S1). Some loss of Sid, even in the absence of gpN cleavage and capsid expansion, was expected, and had previously been observed upon prolonged storage of P4 procapsids [4]. Excess proteinaceous material observed in the micrographs most likely resulted from Sid that had been lost from the capsids (Figure 1B).

**Figure 2:**
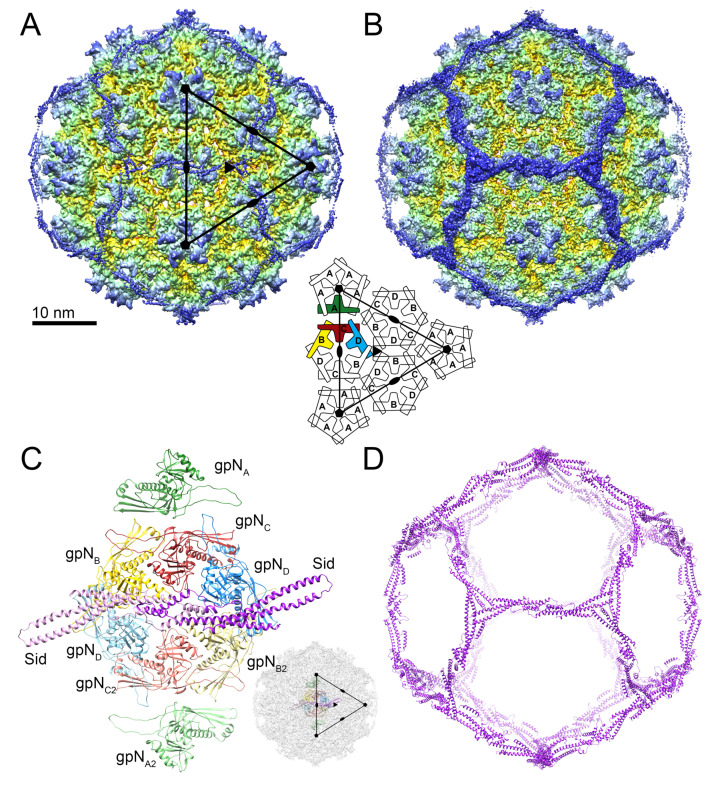
Reconstruction of the P4 procapsid. (**A**) Isosurface representation of the icosahedrally symmetric reconstruction, colored radially from the inside (red) to the outside (blue). The triangle represents one icosahedral face (three asymmetric units); the icosahedral symmetry axes are indicated. The schematic diagram shows the arrangement of gpN subunits in the *T* = 4 lattice. (**B**) Isosurface representation of the asymmetric reconstruction, viewed as in (**A**). (**C**) The atomic model, showing two complete icosahedral asymmetric units, including 8 copies of gpN (A–D and A2–D2) and two copies of Sid. One of the asymmetric units is shown with lighter color. The inset shows the orientation of the model within the capsid. (**D**) Ribbon diagram showing the entire dodecahedral cage formed by Sid.

The scaffold-containing particles were refined with icosahedral symmetry averaging to a resolution of 4.13 Å (gold standard methods, FSC = 0.143 criterion; Figure S1). However, the electron density for the external scaffold in this reconstruction was not sufficiently resolved for atomic model building of Sid (Figure 2A).

Given the known propensity for loss of Sid, we hypothesized that the Sid density in our reconstruction was deteriorated by incomplete occupancy of Sid in some particles or by local variations in the orientation of the scaffolding, resulting from relatively weak interactions with gpN. To rectify this problem, we used a focused asymmetric reconstruction approach (Figure S1): Firstly, a soft mask was generated that encompassed the Sid density surrounding two adjacent three-fold symmetry axes and bridging across a two-fold symmetry axis. Icosahedral symmetry expansion was used to reorient every unique copy of Sid in the dataset into each of the symmetry-related positions inside the soft mask. Masked 3D classification removed particles lacking Sid or with aberrant Sid density in the masked area. The remaining particles were refined with an expanded soft mask and without symmetry averaging, resulting in an asymmetric reconstruction of the P4 procapsid (Figure 2B) at a resolution of 4.19 Å (FSC = 0.143; Figure 1C, Figure S2; Table S1). With alternative masking in Phenix, the map reached a global resolution of 3.91 Å (Figure 1C); however, the local resolution in most of the map was ≈3.5 Å (Figure S2). In the final reconstruction, amino acid side chains were clearly visible throughout the density.

An initial atomic model of gpN was generated using I-TASSER, fitted to all unique locations in the asymmetric reconstruction, and adjusted in *Coot*. No reliable starting model for Sid could be generated by structure prediction, so Sid was modeled de novo beginning with α-helices containing distinctive amino acid side chains. The models were iteratively refined locally with *Coot* and globally with phenix.real_space_refine, including bordering symmetry-related copies to constrain intermolecular interactions. A second, symmetry-related copy of each of the gpN subunits and Sid was added and further refined to represent a disulfide linkage between adjacent Sid monomers (Figure 2C,D). The model and map were consistent to a resolution (FSC = 0.5) of 4.17 Å (Figure 1C; Table S1).

### 3.2. The gpN Capsid Protein

**Figure 3:**
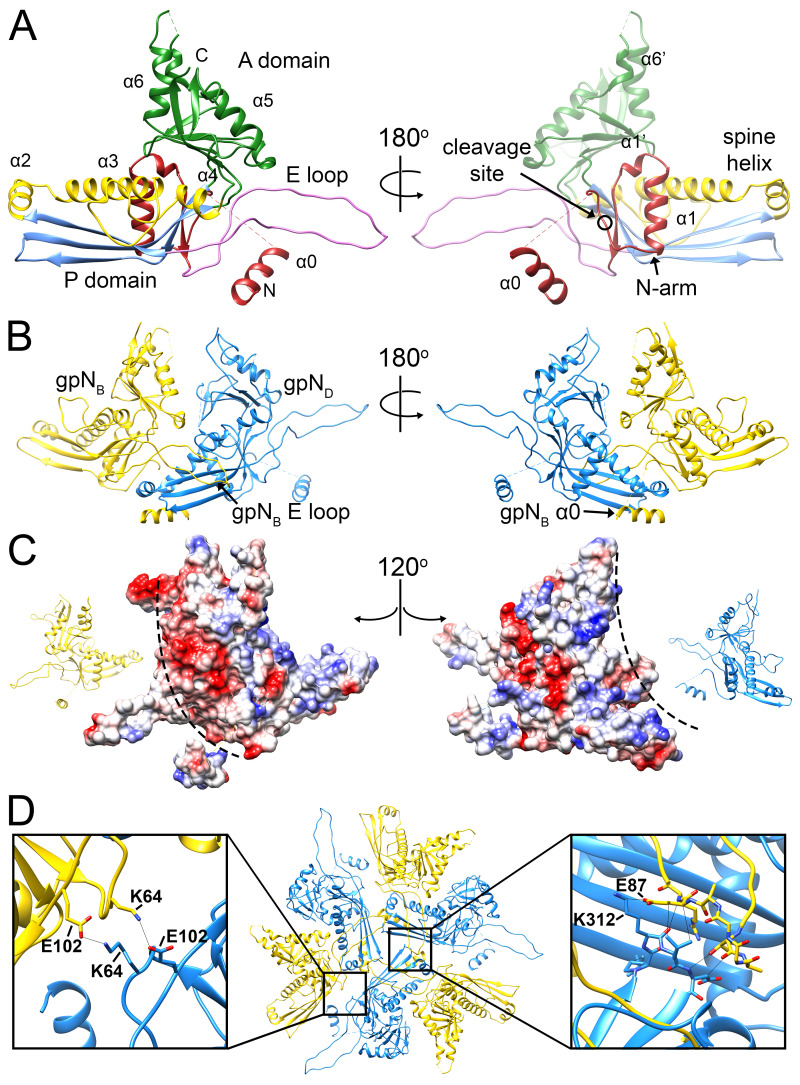
The gpN capsid protein. (**A**) Ribbon diagram of gpN_B_ (the most complete subunit), colored by structural feature: N-arm, red; E-loop, pink; P-domain β-sheet, blue; P-domain α-helices, yellow (α3 is the spine helix); A-domain, green. The right-hand panel is rotated by 180°. Key structural features are labeled, and the gpO cleavage site between residues 31 and 32 is indicated (circle). (**B**) Intra-capsomer interaction between the gpN_B_ (yellow) and gpN_D_ (blue) subunits, showing how the gpN_B_ E-loop overlays the spine helix of the neighboring (gpN_D_) subunit. The right-hand panel is rotated by 180° and shows how the gpN_B_ N-arm and α0 grasp the P-domain of gpN_D_ from underneath. (**C**) Electrostatic surfaces for the gpN_B_ and gpN_D_ subunits from panel B, separated and rotated by 120° in opposite directions, as indicated by the insets. Interaction surfaces of opposite charge are indicated by the dashed lines. (**D**) Inter-capsomeric interaction between three gpN_D_ and their neighboring gpN_B_ subunits related by the icosahedral threefold axis. The insets show details of the interactions between the gpN_D_ and gpN_B_ subunits.

As shown previously [9], the P4 procapsid is organized on a *T* = 4 lattice, resulting in four copies of gpN in the icosahedral asymmetric unit, here denoted by subscripts A–D (and A2–D2 for the second asymmetric unit; Figure 2A,C). The gpN_A_ (green) subunits form the pentamers at the icosahedral fivefold symmetry axes, while two copies each of gpN_B_ (yellow), gpN_C_ (red) and gpN_D_ (blue) form the hexamer at the icosahedral twofold axis (Figure 2A,C). gpN has the expected canonical HK97-like phage capsid protein fold [38], consisting of an N-arm, an E-loop, a P-domain and an A-domain (Figure 3A). The P-domain includes a long “spine” α-helix (α3), and the E-loop extends from the P-domain by ≈45 Å. The A-domain constitutes the protruding part of the capsomers (Figure 3A). The atomic model for the most complete (gpN_B_) subunit includes residues 1–14, 27–253 and 261–346 of the 357 residues encoded by the *N* gene. Some residues were omitted from the other subunits based on variations in local density.

The N-arm includes an α-helix (α1) that folds underneath the P-domain, similar to other procapsid structures [39,40]. Upon capsid expansion, this arm is expected to rotate outward. During a P2 or P4 infection, the trigger for expansion is thought to be cleavage of gpN, during which the first 31 amino acids are removed by the gpO protease [7,13]. In the procapsids analyzed here, which were assembled in the presence of protease-deficient gpO mutant protein, there is no cleavage. A difference map calculated by subtracting density for gpN residues 27–346 and Sid revealed triplets of α-helical densities surrounding the trimeric interfaces between capsomers on the interior of the procapsid (Figure S3A,B). We considered whether this could be a piece of the internal scaffolding protein, gpO. However, the densities only matched the N-terminal 14 residues of the gpN N-arm, based on the observation of side chain densities for Arg6, Phe9, and Tyr12 (Figure S3C). No similar motif could be identified in gpO. These residues comprise an additional α-helix (α0) that had previously been predicted based on sequence analysis [9]. No density corresponding to residues 15–26, connecting α0 to the rest of the N-arm, could be seen in the map.

GpN is organized into pentamers and hexamers on the *T* = 4 lattice (Figure 2). Within the capsomer, the E-loop of one subunit interacts with the P-domain spine helix of the adjacent subunit (Figure 3B). In addition, the α0 helix in the N-arm interacts with the P-domain of the adjacent subunit, allowing the N-arm to wrap around the neighboring spine helix (Figure 3B). The capsomers are also held together by complementary surface charges between subunits (Figure 3C).

The hexamers, which sit on the twofold symmetry axes, have a distinct twofold skew typical of bacteriophage capsids, while the fivefold symmetric pentamers are more tightly packed due to the higher curvature at capsid vertices. This variation is accommodated by flexibility in the E-loop. In gpN_A_, the orientation of the E-loop is shifted by 19° compared to gpN_B-D_ (Figure S4A). Conformational differences in the A domain include the α6–α6′ loop (Figure S4B,C), which is ordered only in gpN_B_, presumably due to contacts with the Sid scaffold (see below). Apart from these differences, the four quasi-equivalent gpN subunits are highly similar (Table S2).

Hexamers and pentamers are held together by trivalent interactions at the icosahedral and quasi-threefold axes. At the icosahedral threefold axes, the P-domains from three gpN_D_ subunits form a ring-like structure together with the E-loops from the adjacent gpN_B_ subunits (Figure 3D). The E-loops reach across their neighboring gpN_D_ subunits to make contacts (primarily electrostatic) with the gpN_D_ subunits in the next, threefold related hexamer. In addition, there is a quasi-twofold symmetric interaction that involves residues Glu102 and Lys64 (Figure 3D). Similar contacts are made at the quasi-threefold axes between gpN subunits from two hexamers and one pentamer (not shown). Together, these interwoven interactions provide a strong, yet flexible lattice, without an extended “P-loop” as in phage 80α [40], chemical crosslinking as in HK97 [41], or stabilizing decoration proteins as in phage λ [42].

### 3.3. The External Sid Scaffold

**Figure 4:**
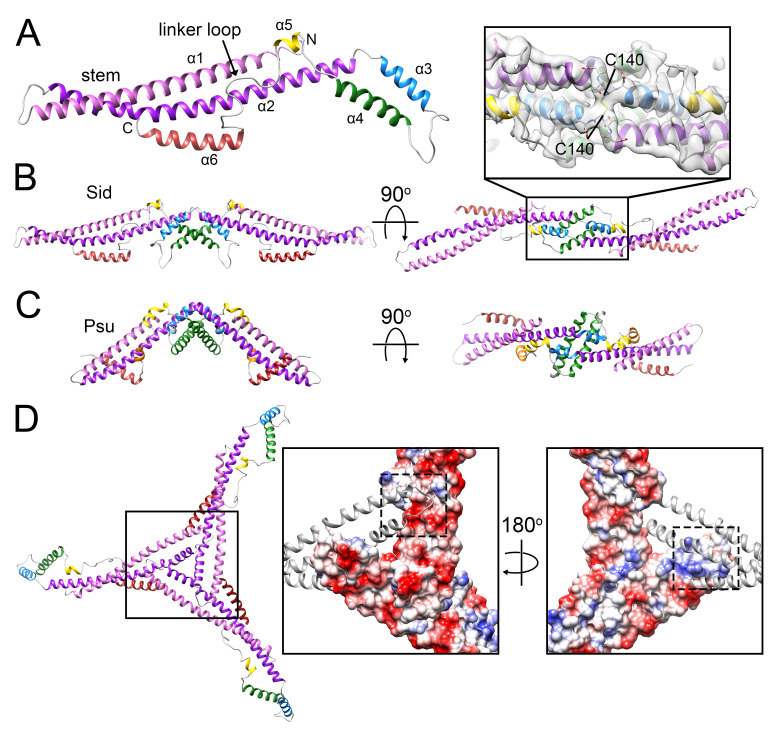
The Sid scaffold. (**A**) Ribbon diagram for one monomer of Sid. Relevant structural features are labeled. (**B**) Ribbon diagram of the Sid dimer, viewed from the side, perpendicular to the twofold axis (left) and from the top, looking down the twofold axis (right). Inset: detail of the twofold interaction that includes the disulfide bond (C140). The map is shown as a transparent gray isosurface. (**C**) The Psu dimer (PDB ID: 3RX6) [45], viewed perpendicular to (left) and down (right) the twofold axis. (**D**) Ribbon diagram of the Sid trimer, viewed down the icosahedral threefold axis. The insets on the right show details of the threefold interaction. Two Sid subunits are shown as electrostatic surfaces, with the third subunit as a ribbon diagram. The left panel shows the view from the outside of the capsid, while the right panel is viewed from the inside. The surfaces that interact are indicated by the dashed squares.

Sixty copies of Sid form a dodecahedral cage surrounding the P4 procapsid, interacting with the underlying capsid shell only at the hexamers, and forming trimeric connections at the icosahedral threefold axes (Figure 2D). Sid is a highly elongated (126 Å), aliform structure, consisting of five extended α-helices interspersed by loops, the longest of which (residues 189–219) contains an additional short helix (Figure 4A). An atomic model was built for residues 7–241 (out of 244). Residues 11–137 form a 100 Å long α-helical coiled-coil (the “stem”) consisting of the two long helices α1 and α2 (Figure 4A). Residues 139–189 form an extensive dimerization interface that includes helices α3 and α4, where the two monomers of Sid interlock in a knot-like structure overlaying the two-fold symmetry axis (Figure 4B). The knot includes a disulfide bond formed by Cys140, which covalently links the Sid subunits in the dimer (Figure 4B). The biological relevance of this disulfide bond is unclear, since disulfides are not expected to form in the *E. coli* cytoplasm [43], and the procapsid does not normally exist as an extracellular entity. A short helix (α5) marks the beginning of a long loop (the “linker” loop; residues 200–219). The density in the linker loop was poorly defined and could not be modeled with confidence. The linker loop connects the main body of the Sid dimer to the C-terminal helix α6 that provides most of the interactions with the gpN proteins underneath.

Upon comparing the Sid structure to other structures in the database, we discovered that the Sid fold is strikingly similar to another P4-encoded protein, the polarity suppressor/decoration protein Psu [22,23] (PDB ID: 3RX6; Figure 4C). Like Sid, Psu binds as a dimer to gpN hexamers on the exterior surface of the capsid, but it is added as a capsid-stabilizing decoration protein after Sid is removed and the capsid has expanded [20,21,23]. The main difference between the two proteins is the longer coiled-coil stem in Sid and the difference in angle between the two subunits (Figure 4C). In Psu, the dimer-forming α4 helices form a tighter junction than in Sid, consistent with the existence of stable Psu dimers in solution [23]. (While Sid appears to form oligomers in vitro, there is no evidence of a stable Sid dimer in solution [44].) The similarity between the two proteins is reflected in their capsid binding mode: Although the existing reconstructions of P4 virions are at very low resolution, Psu dimers can be seen to form bridges across gpN hexamers that are similar to those formed by Sid [20].

**Figure 5:**
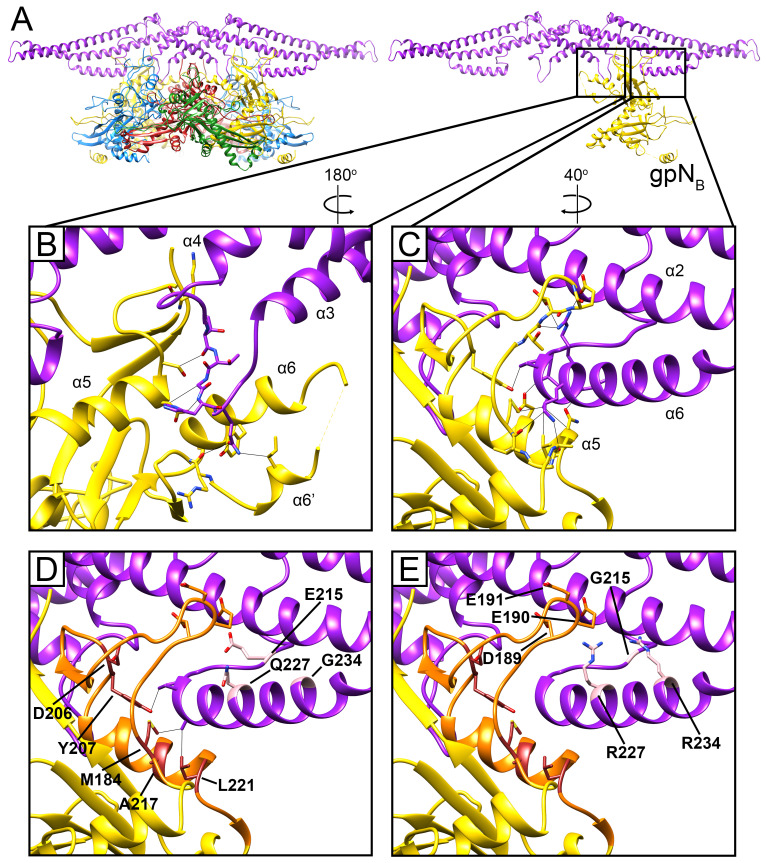
Sid-gpN interaction. (**A**) Ribbon diagram of the Sid dimer (purple) and the underlying gpN hexamer; subunits colored as in Figure 2. In the right panel, only gpN_B_ is depicted. (**B**) Detail of the interaction between the Sid α3–α4 loop and gpN_B_. Residues that make contact are shown in stick representation, and contacts closer than 4 Å are shown as gray lines. (**C**) Interactions between the C-terminal helix α6 of Sid and the gpN_B_ *sir* loop. (**D**) Same view as (**C**). The *sir* loop is shown in orange, and the residues mutated in *sir* and *nms* mutations are labeled in. (**E**) Same view as (**D**), showing how *nms* mutations (E215G, Q227R, G234R) might facilitate novel charged interactions with gpN (residues D189, E190, E191).

In Sid, the elongated stems allow three Sid subunits to come together in an overlapping trimer at the icosahedral threefold axis (Figure 4D). The threefold interface is extensive and characterized by electrostatic interactions between the tip of the stem from one subunit with the coiled-coil region of the threefold-related subunit (Figure 4D). Each Sid monomer forms two sets of contacts with the procapsid, exclusively interacting with the two gpN_B_ subunits in each hexamer (Figure 5). Firstly, Sid residues 161 and 163–165 in the α3–α4 loop of the knot interact with several residues in the gpN_B_ A-domain (Figure 5B), likely stabilizing the alternative conformation of the α6–α6′ loop in gpN_B_ compared to the other gpN subunits (Figure S4B,C). Secondly, residues 219–224 in the C-terminal helix α6 of Sid interact with α5 and the loop preceding it in the A-domain of the symmetry-related gpN_B_ subunit across the hexamer (Figure 5C). In this way, each copy of Sid in the dimer binds to both gpN_B_ subunits in a hexamer, forming a bridge that straddles the hexamer (Figure 5A).

The previously characterized *sir* mutations that disrupt the ability of Sid to redirect capsid assembly are located at five sites in gpN [9,26,27]. All five loci reside within residues 184–221, running from the end of α4 in the P-domain through to the end of α5, which include the region of extensive contacts between Sid and gpN (Figure 5D). We previously denoted gpN residues 192–224 as the “*sir* loop” [9]. Based on our structure, we can expand the definition of the *sir* loop to include gpN residues 183-224, which contain all five known *sir* loci (Figure 5D). Some of the *sir* mutations (M184T, Y207F and L221V/Q) are in residues that are directly involved in contacts with Sid (Figure 5D), and presumably work by directly disrupting these interactions. Other *sir* mutations (D206Y, D206∆ and A217E/Q) might work more indirectly through destabilization of the *sir* loop. In contrast, no *sir* mutations are known that disrupt the interactions between gpN and the Sid α3–α4 loop, suggesting that this interaction is less important or more forgiving than the *sir* loop–Sid α6 interaction.

Unlike the *sir* mutations in gpN, the sites of the *nms* (“super-sid”) mutations in Sid do not contribute directly to the interface between gpN and Sid (Figure 5D). Of the *nms* mutations characterized by Kim et al. [27], *nms7* (E215G) is located in the linker loop, while *nms6* (Q227R) and *nms1* (G234R) are located in α6, but on the opposite side of the helix from the binding site for the gpN *sir* loop. It is therefore unlikely that the *nms* mutations suppress *sir* mutations by directly re-establishing interactions with residues at the *sir* mutation sites. Instead, *nms1* and *nms6*, which most efficiently suppress *sir* mutations and introduce long, positively charged Arg side chains, might establish new contacts with the negatively charged residues Asp189, Glu190, and Glu191 in the *sir* loop (Figure 5E). This would circumvent disruption of the binding interface with the rest of the *sir* loop and allow Sid to bind to gpN regardless of the *sir* mutations. This would be consistent with the observed allele independence of the *nms* mutations [27]. For *nms7*, the change from Glu to Gly would introduce greater flexibility in the linker loop, which might allow the α6 helix to change its orientation to accommodate binding to the *sir* loop in a different manner.

## 4. Discussion

Based on genomics and structural analysis, it is clear that all tailed phages are evolutionarily related. The most striking example of this is the conserved capsid fold across all members of the *Caudovirales* observed to date [38]. Other major structural proteins, like the major tail protein, the portal (connector) protein, and certain baseplate proteins are also highly conserved [46,47,48]. Presumably, this relatedness extends to scaffolding proteins as well; however, structural information on scaffolding proteins is still scarce. So far, the only (partial) scaffolding protein structures known are gp7 from *Bacillus* phage ϕ29 [49], the capsid binding domains of the scaffolding proteins of P22 [39,50] and 80α [40], and the CpmB protein of SaPI1 [40,51]. (The structure of the external scaffold of ϕX174, a member of the *Microviridae*, has also been determined, but these viruses are structurally and phylogenetically distinct from the *Caudovirales* [2,52].) No clearly defined “scaffolding fold” has emerged from these studies. Nevertheless, there are commonalities between all these proteins: all are almost entirely α-helical, and typically incorporate at least one helix-turn-helix motif. They generally form dimers or higher oligomers, most likely by forming α-helical bundles like those seen in ϕ29 gp7 and SaPI1 CpmB [49,51].

P4 is not considered directly related to the *Caudovirales*, but appears to be an independent MGE that has acquired some phage-like functions horizontally, allowing it to take advantage of a phage for its dissemination [15,16]. The Sid scaffolding protein has no known analog in phages. Nevertheless, like the internal scaffolding proteins described above, Sid is an entirely α-helical protein that forms long parallel bundles, most likely as a result of convergent evolution. Perhaps this kind of structure is favorable for scaffolding purposes by providing maximum flexibility combined with high stability. Indeed, this design is also found in unrelated proteins that serve similar roles, such as the F-BAR proteins involved in imposing curvature on vesicles [53].

The strong similarity between Sid and Psu was unexpected. The *sid* and *psu* genes are both encoded within the same operon in the P4 genome, separated by the gene δ [17]. Although the two proteins share only about 17% sequence identity, the program HHpred [54] correctly predicted the similarity with high probability (E-value of 9 × 10^−35^). It thus seems likely that the two genes arose by duplication followed by divergent evolution. If so, what was the function of the protein encoded by the progenitor gene? One possibility is that the polarity suppression function of Psu came first, while capsid binding was a secondary function that was subsequently repurposed by Sid for capsid size redirection. This model is consistent with the view of P4 as primarily a plasmid replicon, which would have had no particular need for capsid binding proteins until it evolved the ability to take advantage of a helper phage like P2. Furthermore, the capsid stabilizing function of Psu is not essential for P4 viability under normal conditions [21]. On the other hand, Psu only binds to small capsids formed in the presence of Sid, suggesting that the Sid-encoded size redirection function must have preceded the capsid binding function of Psu during the evolution of P4. There are no other structures of polarity suppressor proteins, and it is not known whether the Sid/Psu fold is found outside the context of P4 elements.

In general, assembly of phage capsids is thought to proceed through the addition of monomers or small oligomers via a number of energetically equivalent paths [55,56]. How does Sid alter these pathways to redirect the procapsid assembly pathway? Both P2 and P4 capsids are made up of essentially identical hexamers and pentamers [9]. The main difference between a *T* = 4 and a *T* = 7 shell is the angle between the capsomers. We recently described a similar system from *Staphylococcus aureus*, in which the “pirate” element *S. aureus* pathogenicity island 1 (SaPI1) alters the capsid of its helper bacteriophage, 80α, from a *T* = 7 to a *T* = 4 structure [40]. In this case, the size change is caused by an alternate internal scaffolding protein, CpmB, which competes with the cognate scaffolding protein for a binding site on the capsid protein. CpmB alters the angle between capsomers so that the shell has higher curvature. In the more highly curved *T* = 4 shell there is only room to incorporate a pentamer once a trimer of hexamers has formed, whereas in the *T* = 7 shell, the flatter curvature allows for incorporation of one more hexamer [40].

**Figure 6:**
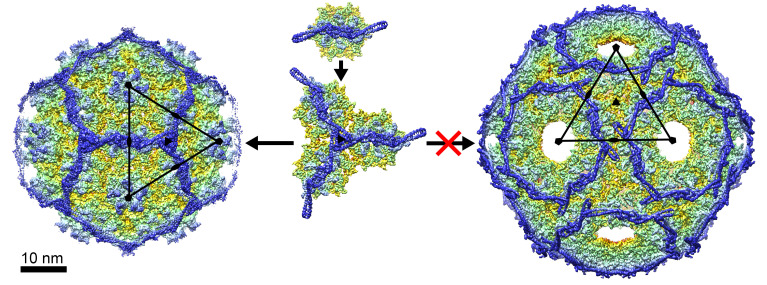
Model for the Sid-induced capsid redirection. Initially, Sid dimers interact with gpN hexamers, forming a Sid-gpN complex (**top**). Once Sid trimerizes and forms a Sid-gpN trimer complex (**middle**), continued growth of the shell is only compatible with formation of a *T* = 4 lattice (**left**). If the Sid-gpN complexes were to assemble into a *T* = 7 lattice (**right**), trimerization of Sid would be impossible, leading to a highly unfavorable configuration of Sid.

Sid works by a different mechanism. The Sid protomers bind gpN hexamers as dimers and tie them together through trimerization of the elongated stems (Figure 6). Since Sid binding is twofold symmetric, only hexamers (and not pentamers) can be captured by Sid dimers. The placement of hexamers on the twofold symmetry axis in a *T* = 4 lattice orients the Sid stems towards the threefold symmetry axis, where they can form trimeric interactions among Sid monomers bound to different gpN hexamers. In contrast, gpN hexamers in the *T* = 7 lattice are oriented such that trimeric interactions of the Sid stems would be impossible (Figure 6). Therefore, Sid must induce the arrangement of hexamers seen in the *T* = 4 lattice by forming these trimeric interactions, thus redirecting procapsid assembly towards the smaller architecture suitable only for packaging of the P4 genome. Consistent with this model, we found that most procapsids do not contain a complete Sid scaffold and exhibit an asymmetric distribution of Sid. This suggests that Sid is only needed at the beginning of the assembly process. Once the curvature has been established, the assembly process is committed to form a *T* = 4 capsid. Alternatively, Sid might assemble and disassemble dynamically as the assembly process progresses.

Like most other members of the *Caudovirales*, P2 also encodes an internal scaffolding protein, gpO. Although gpO is dispensable for capsid assembly in the presence of Sid, gpO is essential for the formation of viable P2 and P4 virions. GpO serves at least two functions in the P2 life cycle: The N-terminal domain (NTD) of gpO (residues 1–141) is a protease that is responsible for the maturational cleavage of gpN and itself, and presumably also gpQ [12]. The C-terminal domain (CTD) of gpO (195–284) is an internal scaffolding protein that is absolutely required for P2 (large) procapsid assembly. GpO is most likely also required for incorporation of gpQ portals—which is obviously essential for DNA packaging—although this has not been proven experimentally. This function might reside in the middle portion of gpO (residues 142–194). The procapsids used in this study were made from a construct expressing full-length, protease-deficient gpO, and SDS-PAGE showed a large amount of gpO protein in the purified procapsids (Figure 1A). We had expected an ordered interaction between the C-terminus of gpO and gpN, similar to what we observed previously in 80α [40]. In spite of this, no density that could be attributed to any part of gpO was apparent in the procapsid reconstructions, presumably due to disorder relative to the capsid. It is worth noting that the procapsids analyzed here did not contain the gpQ portal protein. By comparison with phage ϕ29 [57], it is not unlikely that gpO binds to gpQ during initiation of procapsid assembly, and that this might result in a more ordered distribution of gpO. How this would lead to the assembly of correctly formed *T* = 7 shells, however, is still unclear.

Thanks in great part to the seminal contributions of Michael Rossmann, bacteriophage structural biology today is a vibrant field that employs cutting-edge technology in genomics, biophysics and structural biology, and has found new relevance with the advent of phage therapy and microbiome research. No doubt, high-resolution structures of bacteriophages and phage proteins will continue to yield insights into these important biological entities.

## Supplementary Materials

The following are available online at https://www.mdpi.com/1999-4915/12/9/953/s1, Figure S1. Reconstruction scheme; Figure S2. Local resolution; Figure S3. Difference maps; Figure S4. Differences between gpN subunits; Table S1. Reconstruction and refinement statistics; Table S2. Root-mean-square deviations in gpN.
