# Supplementary material for: Structure of the Capsid Size-Determining Scaffold of “Satellite” Bacteriophage P4
Source: Viruses. 2020 Aug 27;12(9):953. doi: 10.3390/v12090953 (PMC7552001; doi:10.3390/v12090953)
Supplement: Supplementary file 1 [file viruses-12-00953-s001.zip › viruses-914783-supplementary.pdf]

viruses
MDPI

SUPPLEMENTARY INFORMATION

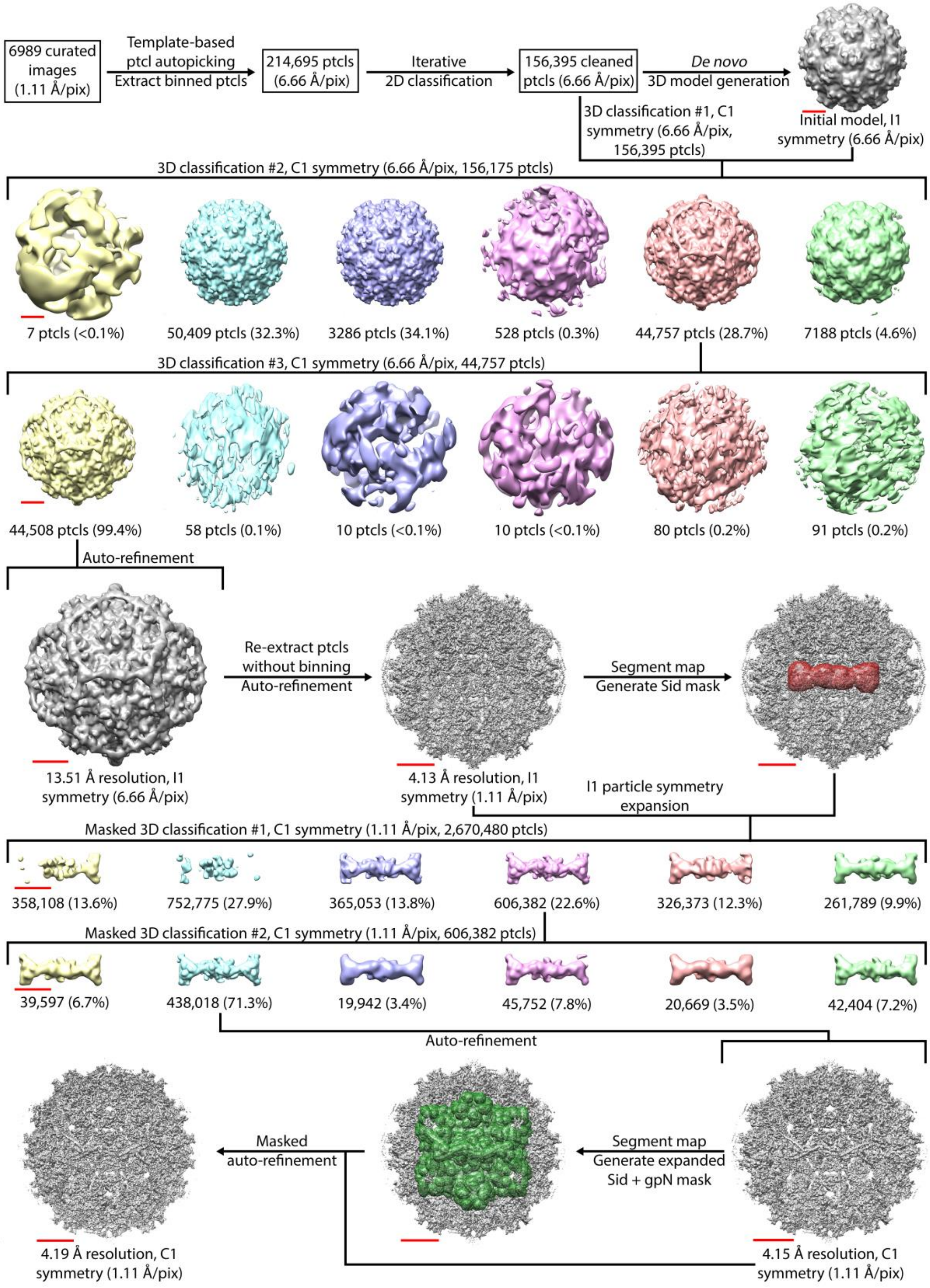

Figure S1. Reconstruction scheme.

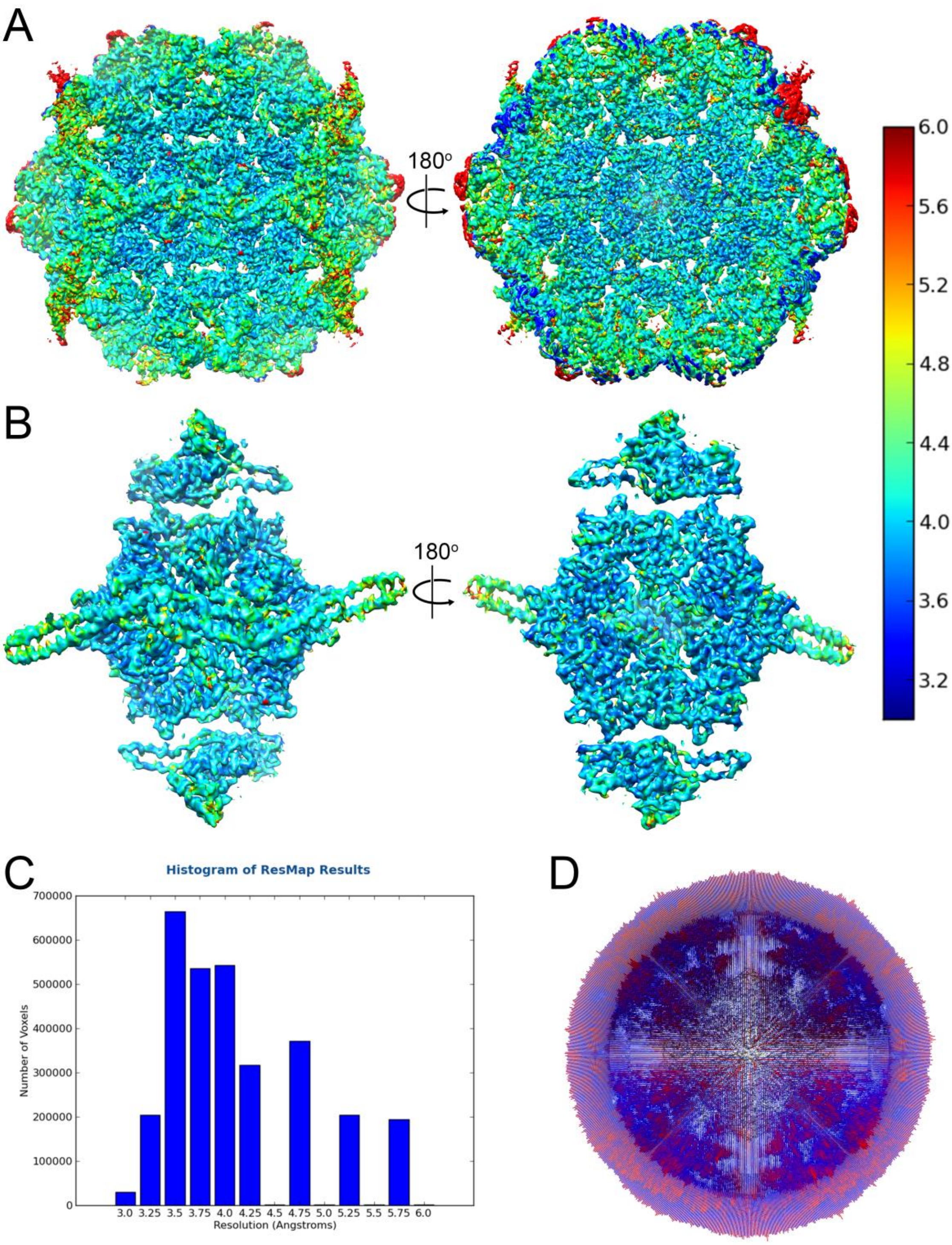

Figure S2. Local resolution. (**A**) ResMap representation of the local resolution of the P4 procapsid reconstruction, colored from blue (≤3Å resolution) to red (≥6Å resolution), showing the portion of the capsid used for refinement. (**B**) Close-up showing the local resolution in two asymmetric units. (**C**) Histogram of distribution of local resolution by voxel. (**D**) Hedgehog representation of the Euler angle distribution.

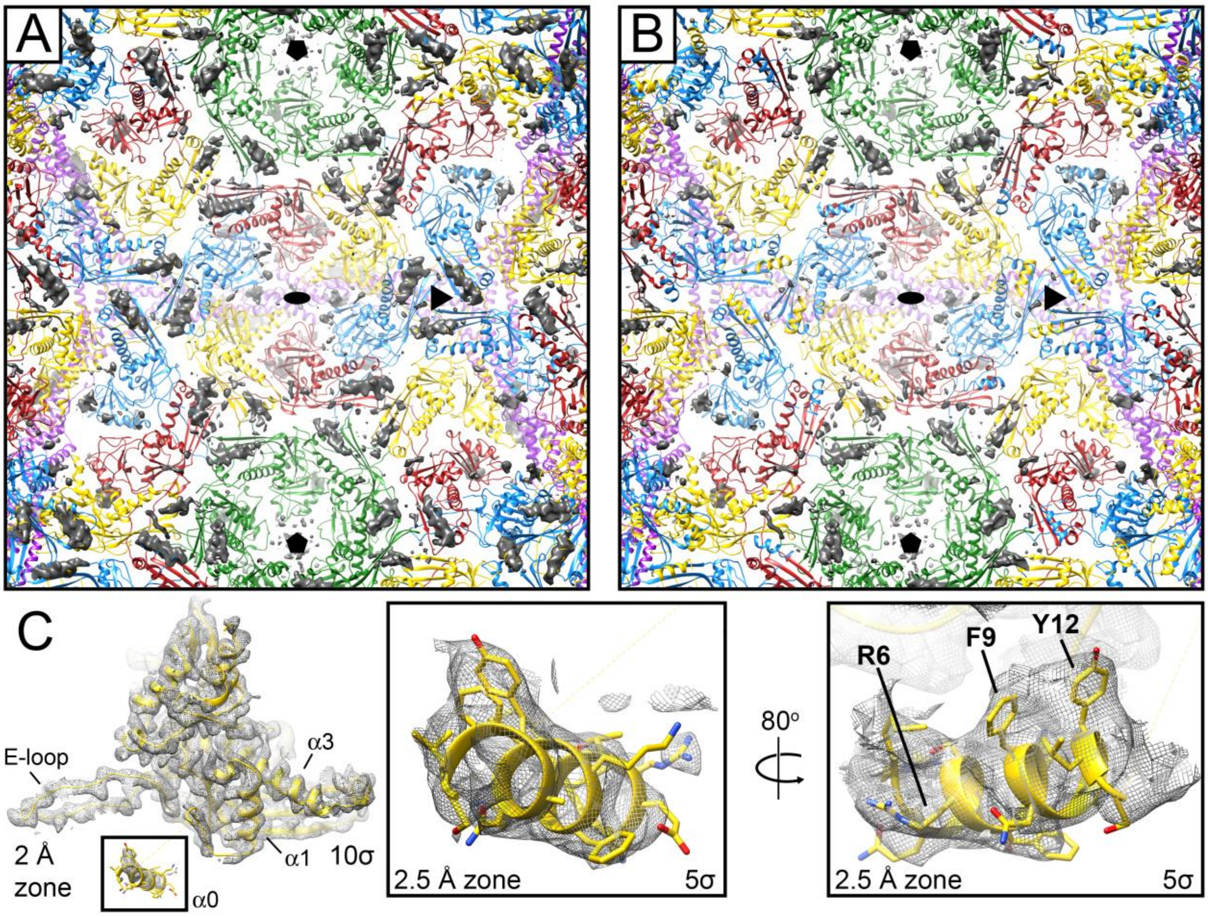

Figure S3. Difference maps. (**A**) View from inside the capsid of a difference map calculated by subtracting the contribution of the modeled residues 27–346 of gpN and 7–241 of Sid, showing the presence of additional density surrounding the threefold axes. GpN and Sid are shown as ribbon diagrams, colored as in Figure 2. (**B**) After modeling gpN residues 1–14 into the densities surrounding the icosahedral threefold axes, only the poorly defined densities around the quasi-threefold axes surrounding the pentamers remain. (**C**) Density (at 10σ cutoff) and atomic model for the gpN_B subunit, showing the additional density due to the α0 helix. Insets: closeup views of the α0 helix density (at 5σ cutoff), showing the densities ascribed to side chains Arg6, Phe9 and Tyr12.

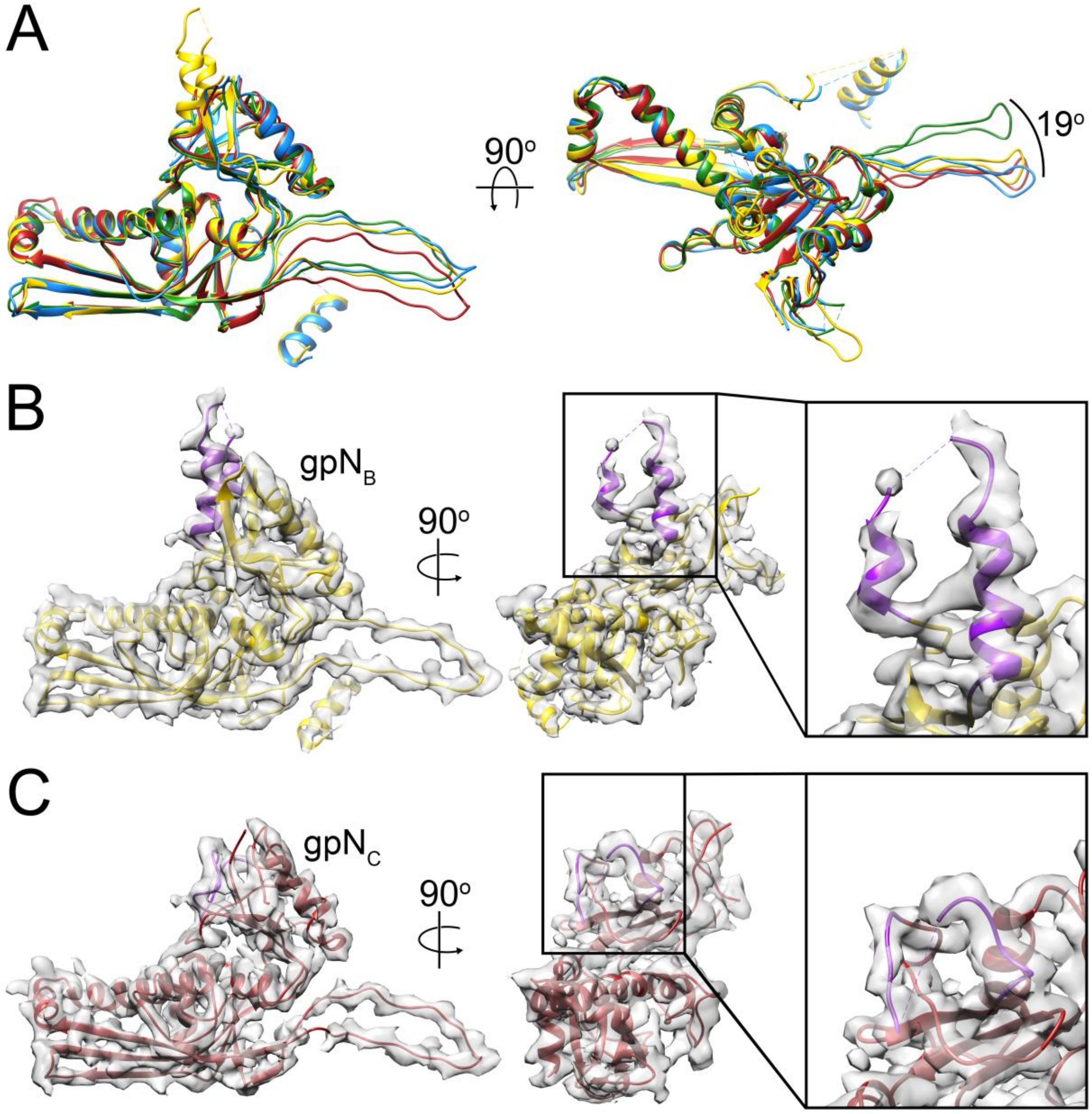

Figure S4. Differences between gpN subunits. (**A**) Superposition of gpN subunits **A–D**, colored as in Figure 2. The 19° rotation of the gpN$_A$ E-loop compared to gpN$_B$–gpN$_D$ is shown most clearly in the 90° rotated panel on the right. (**B**,**C**) Density and atomic model for gpN$_B$ (**B**, yellow), and for gpN$_C$ (**C**, red). The insets on the right show the difference in the loop (purple) that includes the α6-α6′ helices that are only ordered in gpN$_B$, the subunit that interacts with Sid.

Table S1. Reconstruction and refinement statistics.

**Data Collection**	
Microscope	Titan Krios
Detector	Gatan K3
Pixel size (Å)	1.11
Magnification	45000
Electron dose (e^{-}/Å^2)	30
Frames per movie	40
Typical defocus range (μm)	0.5-2.5
**Reconstruction**	
Number of particles	438,018
Software	RELION-3.1
Symmetry	C1
Final resolution (FSC=0.143, Å)	4.19 (RELION); 3.90 (Phenix)
**Atomic Model Refinement**	
Software	phenix.real_space_refine (Phenix v1.18.2); *Coot* v0.8.9.2
Resolution limit (Å)	4.19
Model-map correlation (FSC=0.5, Å)	4.17
Chains	10
Residues	2858
Number of atoms	22758
Protein atoms	22758
Hydrogens	0
Map CC_{box}	0.5
Map CC_{mask}	0.86
RMSD bond length (Å)	0.004
RMSD bond angles (°)	0.593
**Validation**	
Ramachandran plot (%)	
Favored	91.54
Allowed	8.46
Outliers	0
Rotamer outliers (%)	0.16
Clashscore	7.79
EMRinger score	1.48

Table S2. Root-mean-square deviations (RMSD) in gpN. RMSD values (in Å) calculated between equivalent Cα atoms in the four quasi-equivalent gpN subunits A–D. The first set of values include only those Cα atoms that are within 2Å, the second set include all Cα atoms.

RMSD [Å] (included Cα pairs)				
**Cα pairs within 2 Å**				
	gpN_A	gpN_B	gpN_C	gpN_D
gpN_A	0			
gpN_B	0.732 (237)	0		
gpN_C	0.836 (238)	0.837 (230)	0	
gpN_D	0.782 (235)	0.854 (289)	0.809 (239)	0
**All Cα pairs**				
	gpN_A	gpN_B	gpN_C	gpN_D
gpN_A	0			
gpN_B	2.328 (279)	0		
gpN_C	3.261 (274)	2.425 (281)	0	
gpN_D	2.913 (276)	2.379 (307)	2.166 (279)	0
